# Diagnostic Challenges in a Patient with Late-Onset First Manic Episode, Alzheimer’s Disease and Longstanding Depression: A Case Report

**DOI:** 10.1192/j.eurpsy.2026.11582

**Published:** 2026-07-31

**Authors:** A. Čubranić Dominković, G. Rubeša, J. Batur

**Affiliations:** 1 Department of psychiatry, University Hospital Centre Rijeka; 2 Faculty of medicine, University of Rijeka, Rijeka, Croatia

## Abstract

**Introduction:**

Manic episodes arising de novo in late life are rare and often indicate underlying organic pathology. Diagnosis is further complicated by overlapping psychiatric symptoms, neurodegeneration, and psychosocial stress. This report presents a first manic episode in a 66-year-old woman with a 25-year history of depression, recently diagnosed with Alzheimer’s disease and exposed to major psychosocial stress. The case highlights the challenge of distinguishing primary psychiatric illness from organic, iatrogenic, or reactive causes in geriatric psychiatry.

**Objectives:**

- To present a case of late-onset mania in a patient with Alzheimer’s disease and a longstanding depressive disorder

- To explore potential etiological factors contributing to the manic episode
- To highlight the diagnostic challenges related to Alzheimer’s disease, depression, mania, and personality disorders, and emphasizes the importance of a holistic approach in understanding complex psychiatric cases

**Methods:**

A descriptive case report method was used, gathering data through clinical interviews, psychiatric evaluation, neurological assessments, imaging, and collateral information from family and medical records. Mental status examinations were conducted throughout the inpatient stay.

**Results:**

The patient, born in 1959, was hospitalized for a first manic episode marked by elevated mood, psychomotor acceleration, reduced need for sleep, and pressured speech. She had no previous manic or hypomanic episodes but had received outpatient treatment for multiple depressive episodes for over 25 years, starting after her son’s suicide attempt. About four months before admission, her son suffered a serious work injury that left him unable to work—an event she found highly distressing. The manic symptoms began around the same time, suggesting a possible temporal link. She was also diagnosed with Alzheimer’s disease following cognitive decline and neurological assessment; donepezil treatment started shortly before the manic episode. During hospitalization, based on anamnesis and her interpersonal relationships, combined with marked emotional reactivity and longstanding affective instability, a clinical suspicion of underlying personality pathology was raised.

**Conclusions:**

This case illustrates the multifactorial origin of late-onset mania in a patient with Alzheimer’s and chronic depression. Contributing factors include the potential manic effects of donepezil, acute stress from her son’s injury, and cumulative psychological and interpersonal trauma. Diagnosing mania in older adults with neurodegenerative disease requires careful consideration of iatrogenic, psychosocial, and biological factors. This case highlights the need for interdisciplinary assessment of behavioral changes in the elderly, and for heightened awareness of possible mood destabilization following cholinesterase inhibitor treatment.

**Disclosure of Interest:**

None Declared

